# Supramolecular Structure and Functional Analysis of the Type III Secretion System in *Pseudomonas fluorescens* 2P24

**DOI:** 10.3389/fpls.2015.01190

**Published:** 2016-01-05

**Authors:** Ping Liu, Wei Zhang, Li-Qun Zhang, Xingzhong Liu, Hai-Lei Wei

**Affiliations:** ^1^State Key Laboratory of Mycology, Institute of Microbiology, Chinese Academy of SciencesBeijing, China; ^2^Department of Plant Pathology, China Agricultural UniversityBeijing, China; ^3^MOE Key Laboratory of Regional Energy and Environmental Systems Optimization, Resources and Environmental Research Academy, North China Electric Power UniversityBeijing, China

**Keywords:** type III secretion system, PGPR, harpin, MTI, *Pseudomonas fluorescens*

## Abstract

The type III secretion system (T3SS) of plant and animal bacterial pathogens directs the secretion and injection of proteins into host cells. Some homologous genes of T3SS were found also in non-pathogenic bacteria, but the organization of its machinery and basic function are still unknown. In this study, we identified a T3SS gene cluster from the plant growth-promoting *Pseudomonas fluorescens* 2P24 and isolated the corresponding T3SS apparatus. The T3SS gene cluster of strain 2P24 is similar organizationally to that of pathogenic *P. syringae*, except that it lacks the regulator *hrpR* and the *hrpK1* and *hrpH* genes, which are involved in translocation of proteins. Electron microscopy revealed that the T3SS supramolecular structure of strain 2P24 was comprised of two distinctive substructures: a long extracellular, filamentous pilus, and a membrane-embedded base. We show that strain 2P24 deploys a harpin homolog protein, RspZ1, to elicit a hypersensitive response when infiltrated into *Nicotiana tabacum* cv. *xanthi* leaves with protein that is partially purified, and by complementing the *hrpZ1* mutation of pHIR11. The T3SS of strain 2P24 retained ability to secrete effectors, whereas its effector translocation activity appeared to be excessively lost. Mutation of the *rscC* gene from 2P24 T3SS abolished the secretion of effectors, but the general biocontrol properties were unaffected. Remarkably, strain 2P24 induced functional MAMP-triggered immunity that included a burst of reactive oxygen species, strong suppression of challenge cell death, and disease expansion, while it was not associated with the secretion functional T3SS.

## Introduction

In natural systems, plants are attacked continuously by a broad spectrum of pathogens and, at the same time, they are protected by a large number of beneficial microorganisms. For plant pathogenic bacteria such as *Pseudomonas*, *Erwinia*, *Xanthomonas*, and *Ralstonia*, non-flagellar type III secretion system (T3SS) is deployed to secrete and deliver repertoires of effector proteins to suppress innate immunity and cause diseases (Mansfield et al., 2012). The T3SS apparatus, which is referred to as a needle complex, is comprised of three functional modules: (i) a cylindrical basal body that spans the bacterial inner and outer membranes with a presumed central rod to build a channel, (ii) a needle/pilus structure that extrudes from the bacterial outer membrane and functions as a conduit for effector transfer, and (iii) a translocon complex that produces a pore in the host plasma membrane to inject the effectors (Galán and Collmer, 1999; Wei and Collmer, 2012). In recent years, a large variety of structural studies on animal and human bacterial T3SS was conducted. Parts of the system have been described at molecular resolution (Deane et al., 2006; Moraes et al., 2008; Spreter et al., 2009; Schraidt and Marlovits, 2011; Fujii et al., 2012; Bergeron et al., 2013), but few plant-associated bacterial T3SS have been studied, and most of these studies have been in *P. syringae* (Roine et al., 1997; Hu et al., 2001; Jin et al., 2001; Li et al., 2002). In *Pseudomonas syringae* pv. *tomato* DC3000, a pilus is comprised of a major subunit of the T3SS, the HrpA1 protein, which extends to the plant cell (Roine et al., 1997). This pilus is flexible and its length of ∼2 μm is much greater than that of the needles found in animal pathogens (Roine et al., 1997). An *in situ* immunogold labeling was used to visualize the extrusion of the effector and harpin proteins from the tip of the Hrp pilus, which demonstrates that the bacterial pilus can function as a conduit for protein delivery (Jin et al., 2001; Li et al., 2002).

Pseudomonads, which are distributed widely in many natural niches, are an environmentally significant group of bacteria that includes both pathogenic and saprophytic species. Among the pathogenic fluorescent pseudomonads, the importance of the T3SS and its effectors has been well-studied in the opportunistic human and animal pathogen *P. aeruginosa* (Hauser, 2009) and the plant pathogen *P. syringae* (Alfano and Collmer, 2004; Xin and He, 2013). However, in the last 15 years the presence of a T3SS is continuously being reported in a few plant growth-promoting pseudomonads with partial and whole genome sequencing (Preston et al., 2001; Mazurier et al., 2004; Rezzonico et al., 2004, 2005; Mavrodi et al., 2011; Viollet et al., 2011; Barret et al., 2013; Duan et al., 2013; Berendsen et al., 2015). But unlike the plant pathogenic T3SS, the distribution and structure of the plant growth-promoting T3SS are not conserved highly, which results in a poor understanding of its function. For instance, a 20-kb type III gene cluster (*rsp/rsc*) that resembles the *hrp/hrc* locus of *P. syringae* was identified initially in the sugar beet isolate, *P. fluorescens* SBW25, which lacks the harpin-encoding gene, *hrpZ1*, but elicits a hypersensitive response (HR)-like cell death in *Nicotiana clevelandii* (Preston et al., 2001). In Q8r1-96, which is another well-studied *P. fluorescens* strain, a gene-encoding putative harpin-like protein (RspZ) was identified from the T3SS gene cluster, and the overall arrangement of the *rsp/rsc* genes in Q8r1-96 differed from that in *P. fluorescens* SBW25 (Mavrodi et al., 2011). A phylogenic analysis of biocontrol *Pseudomonas* strains based on partial *hrcN* sequences addressed the issue that horizontal gene transfer of the T3SS might take place from pathogenic bacteria to PGPR and that the T3SS apparatus may be maladaptive evolutionarily in some PGPR (Rezzonico et al., 2004). To date, no single T3SS machinery has been isolated from plant growth-promoting rhizobacteria (PGPR).

*Pseudomonas fluorescens* 2P24 is a PGPR strain isolated from wheat take-all decline soil in Shandong Province, China (Wei et al., 2004a). This strain produces several secondary metabolites, such as 2,4-diacetylphloroglucinol (2,4-DAPG), hydrogen cyanide (HCN), and siderophore(s), and it inhibits growth of a range of phytopathogenic fungi (Wei et al., 2004a). The antibiotic 2,4-DAPG was a key determinant in the antibiosis of plant pathogens (Wei et al., 2004b). In addition, strain 2P24 has excellent colonization ability in tomato and cotton rhizospheres to protect against tomato bacterial wilt and cotton rhizoctoniosis, respectively (Wei et al., 2004a). The biocontrol activity of strain 2P24 is regulated by the GacA/GacS two-component system and the quorum sensing regulation system (Wei and Zhang, 2005, 2006). In this work, we report the existence and organization of the T3SS cluster and characterization of a functional harpin-like protein in strain 2P24, and we isolate the T3SS machinery from *P. fluorescens* for the first time. Finally, we demonstrate that strain 2P24 induces strong MAMP-triggered Immunity (MTI), which is not associated with the T3SS.

## Materials and Methods

### Bacterial Strains, Plasmids, and Plant Material

*Escherichia coli* strains were grown in Luria–Bertani (LB) broth at 37°C. *P. fluorescens* and *P. syringae* were grown in King’s medium B (KB) broth (King et al., 1954) at 28°C. Construction of the cosmid library in *E. coli* DH5a was described in Wei and Zhang (2006). A summary of bacterial strains and plasmids is provided in **Table 1**. Antibiotics were used at the following concentrations unless otherwise stated (μg/ml): ampicillin, 100; gentamicin, 20; kanamycin, 50; rifampicin, 50; spectinomycin, 50; and tetracycline, 20. *Nicotiana benthamiana* and tobacco (*N. tabacum* cv. *xanthi*) plants were grown in a greenhouse with 16 h light/8 h dark, 65% humidity, and a temperature of 24°C during daylight and 22°C at night.

**Table 1:** Bacterial strains and plasmids used in this study.

Strain or plasmid	Genotype or relative phenotype	Source
*Escherichia coli*
DH5α	F^-^ *recA1 endA1 hsdR17 supE44 thi-1 gyrA96 relA1*Δ (*argF-lacZYA*)*I169*Φ*80lacZ*Δ*M15*	Sambrook et al., 1989
*Pseudomonas fluorescens*		
2P24	Plant growth-promoting bacterium	Wei et al., 2004a
ΔC	*rscC* deletion mutant	This study
ΔZ1	*rspZ1* insertion mutant	This study
ΔfliC	*fliC* insertion mutant	This study
Pf0-1	Non-T3SS bacterium	Compeau et al., 1988
*P. syringae*		
DC3000	Wild type; Rif^r^	Buell et al., 2003
ΔhopQ1	*hopQ1* deletion mutant of strain DC3000	Wei et al., 2007
plasmids		
pBluescript II SK+	ColE1 origin; Ap^r^	Stratagene
pHSG299	ColE1 origin; Km^r^	TaKaRa
pRK2013	ColE1 replicon with RK2 transfer region, helper plasmid; Km^r^	Figurski and Helinski, 1979
pENTR/SD-TOPO	Entry vector for Gateway cloning; Km^r^, Cm^r^	Invitrogen
pET-22b(+)	Expression vector; Ap^r^	Novagen
pLAFR5	*ori*T cosmid; Tc^r^	Keen et al., 1988
pN11-7	*rsp* genes positive cosmid clone	This study
pN13-41	*rsp* genes positive cosmid clone	This study
pN31-20	*rsp* genes positive cosmid clone	This study
pBS11-7a	pBluescript containing 1.5 kb *Hin*d III fragment from pN11-7	This study
pBS11-7b	pBluescript containing 5.8 kb *Eco*R I fragment from pN11-7	This study
pBS11-7c	pBluescript containing 6.4 kb *Eco*R I fragment from pN11-7	This study
pBS31-20b	pBluescript containing 4.1 kb *Eco*R I fragment from pN31-20	This study
pBS31-20a	pBluescript containing 6.6 kb *Kpn* I fragment from pN31-20	This study
pBS13-41	pBluescript containing 14 kb *Kpn* I fragment from pN13-41	This study
pHSGΔC	pHSG299::*rscC*	This study
pHSGΔZ1	pHSG299::*rspZ1*	This study
pHSGΔfliC	pHSG299::*fliC*	This study
pHIR11	pLAFR3 containing the *P. syringae* pv. *syringae* 61 *hrp-hrc* gene region	Huang et al., 1988
pLN18	pHIR11 derivative with *shcA* and *hopA1* replaced by an *nptII* cassette	Huang et al., 1991
pCPP3297	pLN18 containing an unmarked *hrcC* deletion	Huang et al., 1991
pCPP2274	pHIR11 containing an unmarked deletion *hrpZ1*	Gopalan et al., 1996
pCPP5371	pBBR1MCS containing *avrPto1* promoter, Gateway reading frame B cassette, and codons 2 to 406 of *cya*; Gm^r^, Cm^r^	Oh et al., 2007
pCPP5372	pBBR1MCS containing *avrPto1* promoter, Gateway reading frame B cassette, and *C*-terminal HA tag; Gm^r^, Cm^r^	Oh et al., 2007
pEN-rspZ1	pENTR/SD-TOPO containing full length of *rspZ1*without stop codon	This study
pET22-rspZ1	pET22b expressing *rspZ1*	This study
pET22-hrpZ1	pET22b expressing *hrpZ1_DC3000_*	This study
p5372-rspZ1	pCPP5372 expressing *rspZ1*	This study
p5371-hopQ1	pCPP5371 expressing *hopQ1*	This study
p5372-hopQ1	pCPP5372 expressing *hopQ1*	This study

### DNA Manipulations

Recombinant DNA techniques were performed according to standard protocols (Sambrook et al., 1989). Electroporation was performed using a Bio-Rad GenePulser according to the manufacturer’s protocol (Bio-Rad). Triparental mating was carried out using helper plasmid *E. coli* HB101 (pRK2013) according to the standard protocol. Plasmid DNA preparation and DNA gel extraction were done using the QIAprep Spin Miniprep Kit and QIAquick Gel Extraction Kit, respectively (Qiagen, China). Gateway recombination was conducted with LR clonase II, as recommended by the manufacturer (Invitrogen, China). DNA sequences of pN11-7, pN13-41, and pN31-20 were subcloned into pBluescript using *Eco*R I, *Hin*d III, and *Kpn* I. Subclones were mapped, and selected subclones were sequenced using T3 and T7 primers. Additional sequences were obtained from subclones using specific oligonucleotide primers. All DNA sequencing was done at Invitrogen China. DNA sequences were assembled and analyzed with the sequence analysis software package of the Genetics Computer Group. DNA sequences were deposited in GenBank under the accession number KT582783.

### Construction of the *fliC*, *rspZ1*, and *rscC* Mutants

To make a *fliC* mutant of *P. fluorescens* 2P24, a 0.5-kb fragment of *fliC* gene was amplified by PCR using primers P141 (5′-GCCGGCCTGCAAATCGCTACC-3′) and P655 (5′-ACCTCTACCACGACCAGTCTGC-3′) cloned into the *Sma*I site of pHSG299. The resulting pHSG299 derivative was transformed into *P. fluorescens* 2P24 to produce 2P24Δ*fliC* with kanamycin resistance. The *fliC* mutant was confirmed by the swimming mobility test on KB medium containing 0.3% agar. The *rspZ1* mutation of 2P24 was made using a similar procedure as described above after a 0.5-kb fragment of *rspZ1* gene was amplified using primers P181 (5′- GGTTCGACGGGCGGACAGTCTC -3′) and P681 (5′-GCGCGCCGGGTGCTGGTCCAT-3′). To delete the *rscC* gene from strain 2P24, two 1.1-kb fragments carrying the left and right flanking regions of *rscC* were amplified by PCR using primer pair P2658 (5′-CCGCCCATGAATTCCGCCG-3′) and P3740 (5′-GGTACGGGATCCGCGGACAC-3′) and primer pair P4332 (5′-TCGGGATCCACCGCACGCAAC-3′) and P5548 (5′-CGGCTGCAGGCATCGCCCTG-3′), respectively. Each fragment of the left and right regions was digested with relative restriction enzymes and was cloned into the *Eco*RI and *Pst*I sites of pHSG299. The resulting pHSG299 derivative was transformed into *P. fluorescens* 2P24. The kanamycin resistance colonies were cured of pHSG299. The final kanamycin-sensitive mutants were screened by PCR.

### Isolation of Type III Secretion Machinery from *P. fluorescens* 2P24

The methods used for *P. syringae* (Roine et al., 1997; Jin and He, 2001) and *Salmonella* (Kubori et al., 1998) were modified to isolate the *P. fluorescens* type III secretion machinery. Briefly, a 2 ml aliquot of overnight culture was inoculated into 200 ml KB liquid media, and the bacteria were grown until OD_600_ 0.8. The bacteria collected by centrifugation were suspended in 20 ml of 0.5 M ice-cold sucrose solution containing 0.15 M Tris, 10 mM EDTA (pH 8.0), and 1 mg/ml lysozyme, followed by incubation for 1 h at 4°C. The resulting spheroplasts were lysed by addition of 30% Triton X-100 to the final concentration at 0.1% and incubated for 2 h at 4°C. Then, we added 2.3 ml 100 mM MgSO_4_ and centrifuged at 8000 r/min for 20 min. The supernatant was transferred to a new tube and adjusted to pH 11 with 1 M NaOH, followed by incubation at 4°C. To collect type III complexes, the lysate was subjected to ultracentrifugation at 22000 r/min for 1 h at 4°C. The pellet was then suspended in 20 ml buffer with 0.1 M KCl-KOH (pH 11.0), 0.5 M sucrose, and 0.1% Triton X-100, and centrifuged at 22000 r/min for 1 h at 4°C. We discarded the supernatant and added 15 ml buffer with 10 mM Tris-HCl (pH 8.0), 5 mM EDTA, 0.1% Triton X-100, and then fractionated with 30%(w/v) CsCl density gradient centrifugation at 22000 r/min for 2 h 4°C. The pelleted type III secretion complexes were suspended in a small amount of buffer and examined immediately using a JEOL JEM-1400 Transmission Electron Microscope (TEM).

### Expression and Purification of Harpins

For the expression and purification of soluble harpins, the *hrpZ1*_DC3000_ and *rspZ1* genes lacking stop codons were cloned into pET-22b expression vector, respectively. Derivative pET-22b plasmids produce C-terminal fusions with the His_6_ affinity tag. pET-22b plasmids were transformed into *E*. *coli* BL21(DE3). The expression process was described in a previous report with a slight modification (Kvitko et al., 2007). Single colonies of the expression strains were inoculated into 10 ml LB containing ampicillin and incubated for 12 h with shaking at 37°C. The starter cultures were used to inoculate 100 ml LB-carbenicillin cultures in flasks, which were incubated with shaking at 30°C to an optical density at 600 nm of ca. 0.4. IPTG was added to a final concentration of 1 mM, and the cultures were allowed to incubate for an additional 6 h. Cell pellets were harvested and lysed according to the Qiagen protocol. The suspension was sonicated on ice with a Fisher Scientific 550 sonic Dismembrator. Cleared lysates were harvested at 12,000 rpm for 30 min at 4°C and purified with Ni-nitrilotriacetic acid (NTA) agarose (QIAGEN). Purified harpins were stored at 4°C until use. Proteins were visualized by sodium dodecyl sulfate-polyacrylamide gel electrophoresis (SDS-PAGE), followed by staining with Coomassie brilliant blue. Protein concentrations were determined by the Bradford assay. Harpin preparations were diluted for plant reaction assays. Empty-vector preparations were obtained in parallel with each harpin preparation.

### Western Blot and CyaA Translocation Reporter Assays

HopQ1 protein in cell and supernatant fractions were analyzed essentially as described by Kvitko et al. (2007). Samples were separated by electrophoresis on SDS-PAGE and analyzed by immunoblotting using primary anti-HA mouse monoclonal immunoglobulin G (IgG) antibodies and secondary anti-mouse IgG alkaline phosphatase conjugate antibodies, as described by Kvitko et al. (2007). An adenylate cyclase (CyaA) injection assay was used to test HopQ1 translocation (Kvitko et al., 2007). This assay can determine if a type III effector-CyaA fusion is injected into eukaryotic cells, because the CyaA enzyme is dependent on calmodulin, a protein present in sufficient amounts only inside eukaryotic cells. When a CyaA fusion is injected into plant cells, there is a substantial increase in cAMP, a product of the CyaA-catalyzed reaction. The fresh bacterial cells in 10 mM MgCl_2_ were infiltrated on *N. benthamiana* leaves at 10^8^ cfu/ml. The leaf disks were collected at 12 h with a 1.0-cm-diameter cork borer. The cyclic AMP (cAMP) levels were determined by using a Correlate-EIA cAMP immunoassay kit according to the manufacturer’s instructions (Enzo, USA).

### Phenotypic Characterization of the *rscC* Mutant

The *rscC* mutant was compared to the parental strain 2P24 for production of the antibiotic 2,4-DAPG, HCN, exoprotease, and siderophores, as described previously (Wei et al., 2004a; Wei and Zhang, 2006). Antibiosis detections of strain 2P24 and the *rscC* mutant were performed on a PDA plate with *Rhizoctonia solani* and *Ralstonia solanacearum* as target pathogens. Plant protection experiments against tomato bacterial wilt were carried out in a greenhouse according to the method of Wei et al. (2004a).

### Assay for Reactive Oxygen Species (ROS)

For ROS measurements, *N. benthamiana* leaf tissue was inoculated with bacterial strains at 10^8^ cfu/ml. At 15 h post-inoculation, leaf disks (0.5 cm diameter) were excised and placed into wells of 96-well plates pre-supplied with 100 ml of sterile water, and then 100 ml of 0.5 mM L-012 (Wako, Japan) in 10 mM morpholinepropanesulfonic acid–KOH buffer (pH 7.4) was added. The intensity of ROS generation was determined according to Oh et al. (2010).

### Challenge-Inoculation HR and Assays for Bacterial Colony Development

The fresh *P. fluorescens* bacterial cells in 10 mM MgCl_2_ were infiltrated on *N. benthamiana* leaves at 10^8^ cfu/ml. *P. syringae* DC3000 was challenge-inoculated 6 h later at 10^7^ cfu/ml to overlap partially with the *P. fluorescens* pretreated area. After 48 h, leaves were photographed. To observe the suppression of inhibition of the growth of the challenge-inoculated virulent pathogen, the *P. fluorescens* strains were inoculated at 10^8^ cfu/ml into *N. benthamiana*, then 6 h later, *P. syringae* DC3000Δ*hopQ1* was challenge-inoculated at 10^5^ cfu/ml on the edge of the pre-infiltrated area to produce an overlapped area and a non-overlapped area. Bacterial populations of *P. syringae* DC3000Δ*hopQ1* in the overlapped area were assessed at 0 and 4 days post-inoculation. Each experiment was repeated three times.

## Results

### Cloning and Sequence Analysis of the *P. fluorescens* 2P24 T3SS Gene Cluster

**FIGURE 1:**
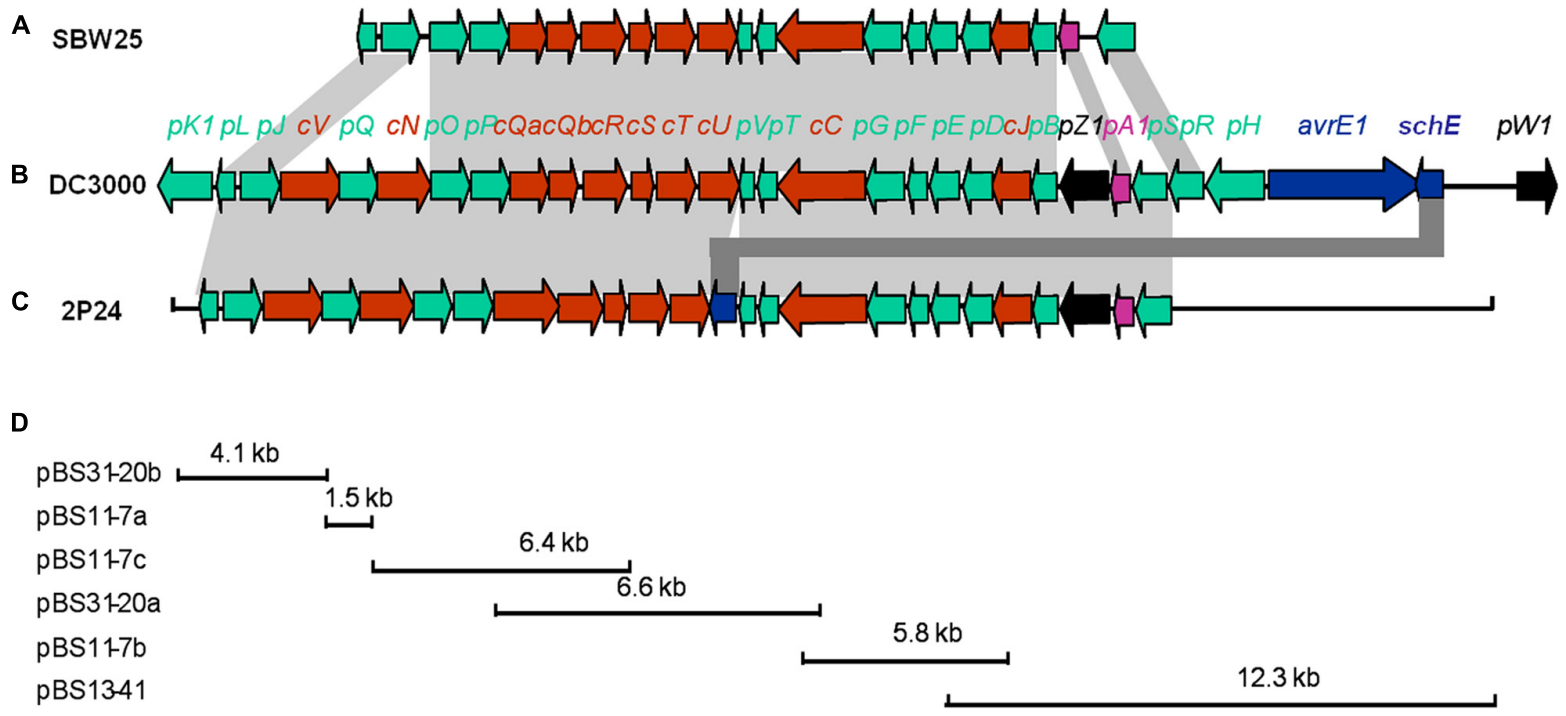
**Genetic organization of the *rsp/rsc* gene cluster of *P. fluorescens* 2P24 **(C)** and comparison with the *hrp/hrc* gene clusters of *P. fluorescens* SBW25 **(A)** and *P. syringae* pv**. ***tomato*** **DC3000 (B)**. Predicted open reading frames and their orientation are shown by large arrows. Conserved *rsc/hrc* genes are shown as red arrows, conserved *rsp/hrp* genes as green arrows, and harpin proteins as black arrows. Additional ORFs are shown as blue arrows. Homologous genes are connected with gray shading. Full gene names within *rsc/rsp* and *hrc/hrp* clusters were shortened and are indicated by a single letter preceded by “*c*” or “*p*”. **(D)** The cloning procedures and subclones contain *rsp/rsc* gene fragments. For details, see **Tables 1** and **2**.

Three cosmid clones, pN11-7, pN13-41, and pN31-20 were isolated from a genomic library of *P. fluorescens* 2P24 using primers P1280 (5′-AACCAGCCGGCKGTSATGA-3′) and P1680 (5′-AGGATGAAGACSCGYTCGCG-3′) designed from *hrcC* homologs. Subsequent mapping and sequencing of the subclones of these cosmids delimited a 30 kb cluster of TTSS-related genes (**Figure 1**). A total of 25 predicted open reading frames (ORFs) were identified and assigned the gene names *rsp* (rhizosphere-expressed secretion gene) and *rsc* (*rsp* conserved) according to the non-pathogenic *Pseudomonas* T3SS convention published previously (Preston et al., 2001; Mavrodi et al., 2011).

The arrangement and orientation of the *P. fluorescens* 2P24 T3SS gene cluster bore a strong similarity to that of *P. syringae*, which has been studied extensively (Huang et al., 1988). It was reported that the *hrp* genes encoding the TTSS machinery in *P. syringae* are the conserved center region (CCR) of a tripartite pathogenicity island that includes exchangeable (EEL) and conserved (CEL) effector loci (Alfano et al., 2000). We did not find any corresponding EEL and CEL loci from the limited sequences of both sides of the 2P24 T3SS cluster. Specifically, we observed that the ORFs that flanked a 9.0 kb upstream region of the *hrpS* gene are similar to proteins unlikely to be linked to type III secretion (**Figure 1A**). The presence of T3SS-like genes has been reported recently in a few non-pathogenic fluorescent pseudomonads (Preston et al., 2001; Rezzonico et al., 2004; Mavrodi et al., 2011), in which T3SS-like genes of *P. fluorescens* Q8r1-96 share the greatest similarity with strain 2P24 (**Table 2**). Overall, it appears that T3SS of strain 2P24 has six principal features:

**Table 2:** Comparison of *rsp/rsc* and *hrp/hrc* cluster proteins in *P. fluorescens* and *P. syringae.*

ORFs	Length of predicted peptides(a.a.) in strain 2P24	Length of predicted peptides(a.a.) in the following strains/percentage identity to that of strain 2P24			Predicted function
		SBW25	Q8r1-96	DC3000	
*pL*	183	183/51	197/98	184/44	RNA polymerase sigma factor; cytoplasmic
*pJ*	380	196/36	375/96	368/36	Type III secreted protein for translocation of effectors
*cV*	695	–^1^	699/63	695/96	Inner membrane associated protein
*pQ*	268	–	297/86	330/32	Similar to FliG, a cytoplasmic protein regulating flagellar biogenesis
*cN*	452	–	452/97	449/69	Inner membrane associated protein, soluble components, ATPases
*pO*	139	148/24	139/90	148/30	Protein of the export apparatus, cytoplasmic, chaperone-like activity
*pP*	189	152/38	189/83	189/28	Required for elicitation of HR and translocation and secretion of AvrPto1
*cQ*	368	337/41	367/87	238+137/37+62^2^	Inner membrane associated protein
*cR*	217	217/68	217/98	200/72	Inner membrane associated protein
*cS*	84	87/71	84/100	88/70	Inner membrane associated protein
*cT*	265	262/59	265/97	264/54	Inner membrane associated protein
*cU*	366	365/58	366/96	359/54	Inner membrane associated protein
*cE*	149	–	149/93	131/49	Type III chaperone ShcE
*pV*	125	117/42	123/94	119/39	Negative regular of *hrp* expression; cytoplasmic
*pT*	69	67/37	69/99	67/50	Accessory protein, outer-membrane associated protein
*cC*	716	713/50	716/96	699/49	Outer-membrane associated protein
*pG*	145	130/31	145/90	143/30	Suppressor of the negative regulator HrpV mediated
*pF*	74	71/22	74/99	74/38	Required for elicitation of HR and translocation and secretion of AvrPto1
*pE*	197	191/31	197/87	193/30	Required for elicitation of HR
*pD*	196	193/33	196/87	176/40	Required for elicitation of HR and translocation and secretion of AvrPto1
*cJ*	295	271/62	293/94	268/58	Putative connectors of the secretion apparatus across the periplasm
*pB*	124	121/29	124/92	124/43	Required for elicitation of HR and translocation and secretion of AvrPto1
*pZ1*	309	–	309/92	370/31	Harpin, type III secreted protein
*pA1*	69	63/22	69/96	113/35	Structural component of pilus, type III secreted protein
*pS*	312	–	307/98	302/59	Transcriptional regulator, cytoplasmic

*^1^The dashes show that there are no homologs present in *P. fluorescens* SBW25*.

*^2^The *hrcQ* gene is segmented into two genes, *hrcQa* and *hrcQb*, in *P. syringae**.

*SBW25, *P. fluorescens* SBW25; Q8r1-96, *P. fluorescens* Q8r1-96; DC3000, *P. syringae* pv. *tomato* DC3000*.

(i) A protein showing 31% identity to HrpZ1 harpin, which resides between HrpA1 and HrpB in the *hrpJ* operon of *P. syringae* DC3000, was identified from the same position in *P. fluorescens* 2P24. A HrpZ1 homolog was found also in Q8r1-96, which is another non-pathogenic *P. fluorescens* (Mavrodi et al., 2011); it was not found in *P. fluorescens* SBW25 (Preston et al., 2001) or *P. fluorescens* F113 (Redondo-Nieto et al., 2013), which are well-known PGPR.
(ii) In strain 2P24, a homolog of the SchE protein is present between HrpV and HrcU, but neither insertion sequences nor duplicated regions were detected around the flanking sequences of the *schE* gene.
(iii) The *P. fluorescens* 2P24 cluster carries an intact *hrpV* operon (*hrpJ, hrcV, hrpQ, hrcN)*, which is significantly absent from *P. fluorescens* SBW25, but is very important for secretion of T3SS substrates in phytopathogenic *Pseudomonas* (Huang et al., 1988; Alfano and Collmer, 1997).
(iv) The *P. fluorescens* 2P24 cluster harbors only a single response regulator homolog, *rspS*, which is one of the two regulators (*hrpS* and *hrpR*) that are 64% identical to that in *P. syringae* DC3000 (Grimm et al., 1995; Deng et al., 1998); this is the same homolog that is found in *P. fluorescens* Q8r1-96 (Mavrodi et al., 2011), but different than the homolog found in *P. fluorescens* SBW25 (Preston et al., 2001).
(v) In strain 2P24, *hrcQ* is an integrated gene, but it is segmented into two genes, *hrcQa* and *hrcQb*, in *P. fluorescens* SBW25 (Preston et al., 2001) and *P. syringae* (Huang et al., 1988).
(vi) Strain 2P24 has all nine *hrc* homologs, which show 58.6% identity to that of phytopathogenic bacteria, but *hrp* homologs show 37.7% identity. This result indicated that the T3SS component genes are more conserved than the genes that are associated with HR and pathogenicity.

### Isolation of T3SS Supramolecular Machinery from *P. fluorescens* 2P24

**FIGURE 2:**
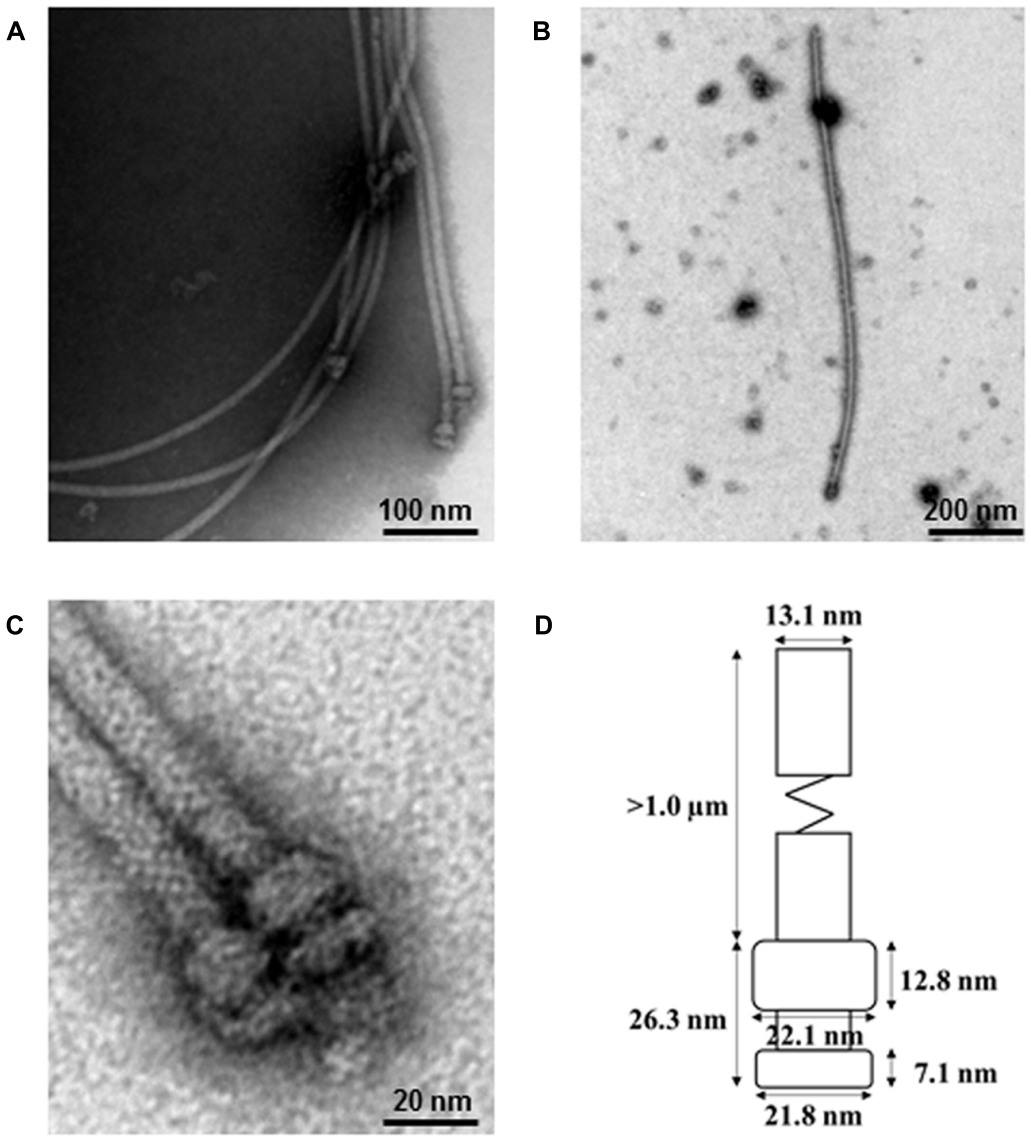
**Electron micrograph of purified type III secretion complexes from *P. fluorescens* 2P24. (A,B,C)** Purified type III secretion complexes at different magnifications. The sample was obtained from the lower fraction of a 30% CsCl gradient centrifugation and observed by TEM. Scale bars as indicated in the images. **(D)** Proposed size of the type III secretion complex of *P. fluorescens* 2P24. The size of each portion was measured based on the 20 best preserved type III secretion complexes.

To identify the *P. fluorescens* type III secretion machinery, we attempted to extract the pilus-like structures from strain 2P24 by a modified method. To prevent the interference of flagella and to facilitate isolation of the T3SS machinery, we first made a non-flagellated mutant by knocking out the *fliC* gene and, therefore, eliminated their swimming ability on the agar plates (Supplementary Figure S1). The *fliC* mutant of strain 2P24 grew to log phase and were shocked osmotically and purified further by 30% (W/V) CsCl density gradient centrifugation. The components of the type III machinery were pelleted by ultracentrifugation, and their structure and morphological features were examined by TEM. The secretion machinery exhibited a pilus-like structure with cylindrical symmetry (**Figure 2**). Individual components of the machinery that were enlarged on electron micrographs indicated that the basal part was comprised of two rings. To estimate the size of each portion, 20 purified type III complexes were measured by TEM. The pilus appendages were curved and they fragmented easily during sampling as reported by Roine et al. (1997) for *P. syringae*. Therefore, the length of the pilus could not be measured accurately. But it would longer than 1 μm and its diameter was approximately 13.1 nm. The length of the basal portion was 26.3 nm. The upper rings were estimated to be 22.1 nm in diameter and 12.8 nm in thickness. The lower rings were similar in diameter to the upper rings, but they were half as thick as the upper rings. To our knowledge, this is the first report that documents the proposed supramolecular structure of the type III T3SS secretion machinery for *P. fluorescens* (**Figure 2D**).

### *P. fluorescens* 2P24 Elicits HR in Tobacco Leaves with the Help of Cosmid pCPP2274, but not pLN18

**FIGURE 3:**
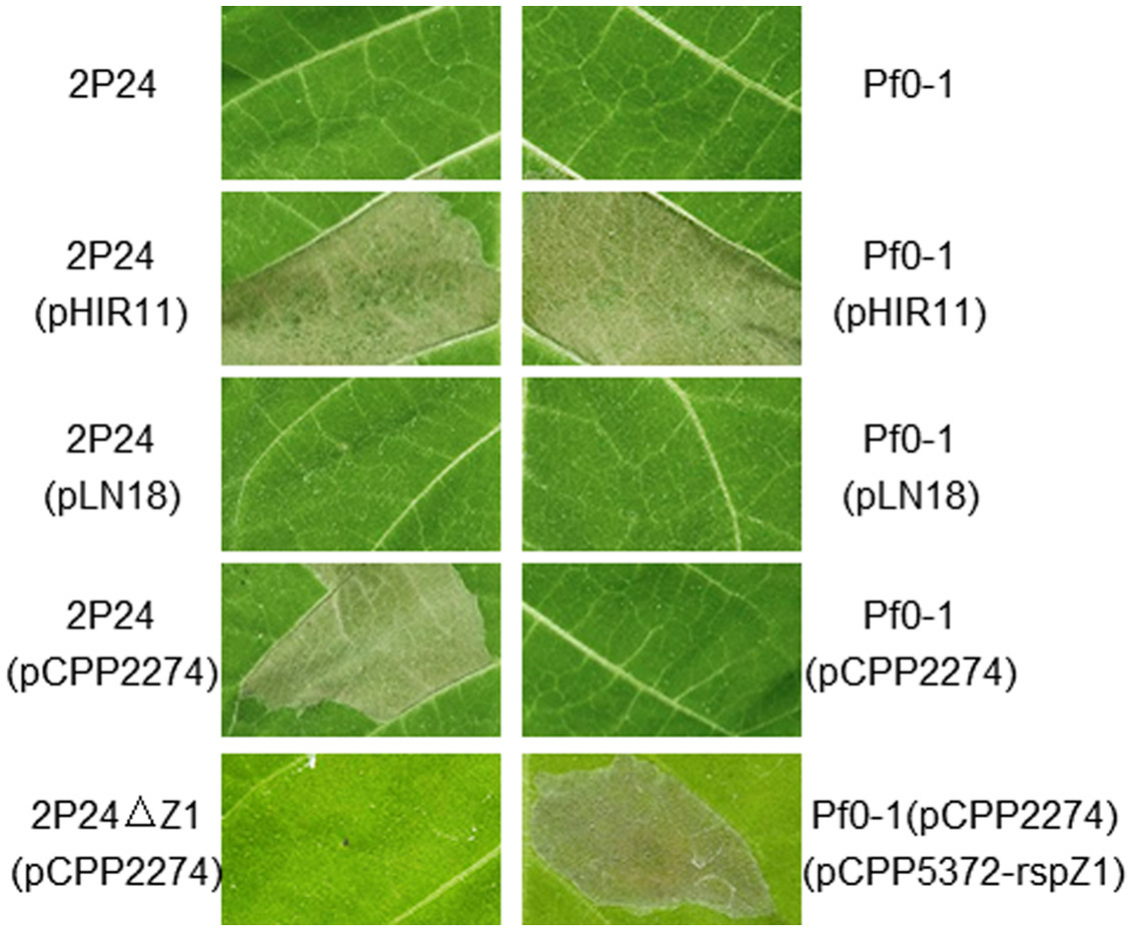
**Elicitation of the HR by *P. fluorescens* constitutively expressing pHIR11 and its derivatives**. Leaf panels of tobacco (*N. tabacum cv. xanthi*) were infiltrated with bacterial suspensions at a concentration of 10^8^ cfu/ml using a blunt-ended syringe. Cell death was evaluated and representative leaves were photographed 48 h after inoculation. The experiment was repeated three times with similar results.

*P. fluorescens* 2P24 does not elicit a HR or any pathogenic symptom when infiltrated into a range of plant leaves such as tobacco, tomato, soybean, rice, and *Arabidopsis thaliana* at inoculum levels up to 10^8^ cfu/ml. No significant increase in bacterial population levels was observed in 7 days after infiltration of *P. fluorescens* 2P24 into plant leaves (Wei et al., 2004a). To determine the potential function of the T3SS components in *P. fluorescens* 2P24, a T3SS cosmid named pHIR11 and its derivatives were used to combine with strain 2P24. *P. fluorescens* 2P24 that carried the *hrp/hrc* cluster of *P. syringae* pv. *syringae* 61 on the cosmid pHIR11 elicited an HR in tobacco at 10^8^ cfu/ml, as was observed for *P. fluorescens* Pf0-1, which is a non-T3SS *Pseudomonas* strain that was used as a control strain (Compeau et al., 1988), (**Figure 3**). The strain of *P. fluorescens* 2P24 that carried pLN18, a derivative of pHIR11 with an insertion mutation in the avirulent *hopA1* gene (Huang et al., 1991), was unable to elicit a HR, as observed for *P. fluorescens* Pf0-1. This indicated that there is no HopA1 homolog in *P. fluorescens* 2P24 similar to that in strain Pf0-1. However, pCPP2274, a derivative of pHIR11 with a mutation in the *hrpZ1* gene, enabled strain 2P24, but not Pf0-1, to elicit a strong HR in tobacco leaves. Therefore, we postulate that the HrpZ1 homolog, RspZ1 of strain 2P24, would complement a harpin deficiency in pCPP2274. We then made a *rspZ1* mutant of strain 2P24, 2P24ΔZ1, and introduced pCPP2274 into this mutant. As expected, 2P24ΔZ1 with pCPP2274 failed to trigger HR in tobacco, and a plasmid-born *rspZ1* in Pf0-1(pCPP2274) recovered HR (**Figure 3**). This indicated that RspZ1 of *P. fluorescens* 2P24 has the same function as HrpZ1 in plant pathogens.

### Partially Purified RspZ1 from Strain 2P24 Can Elicit Cell Death in Tobacco Leaves

**FIGURE 4:**
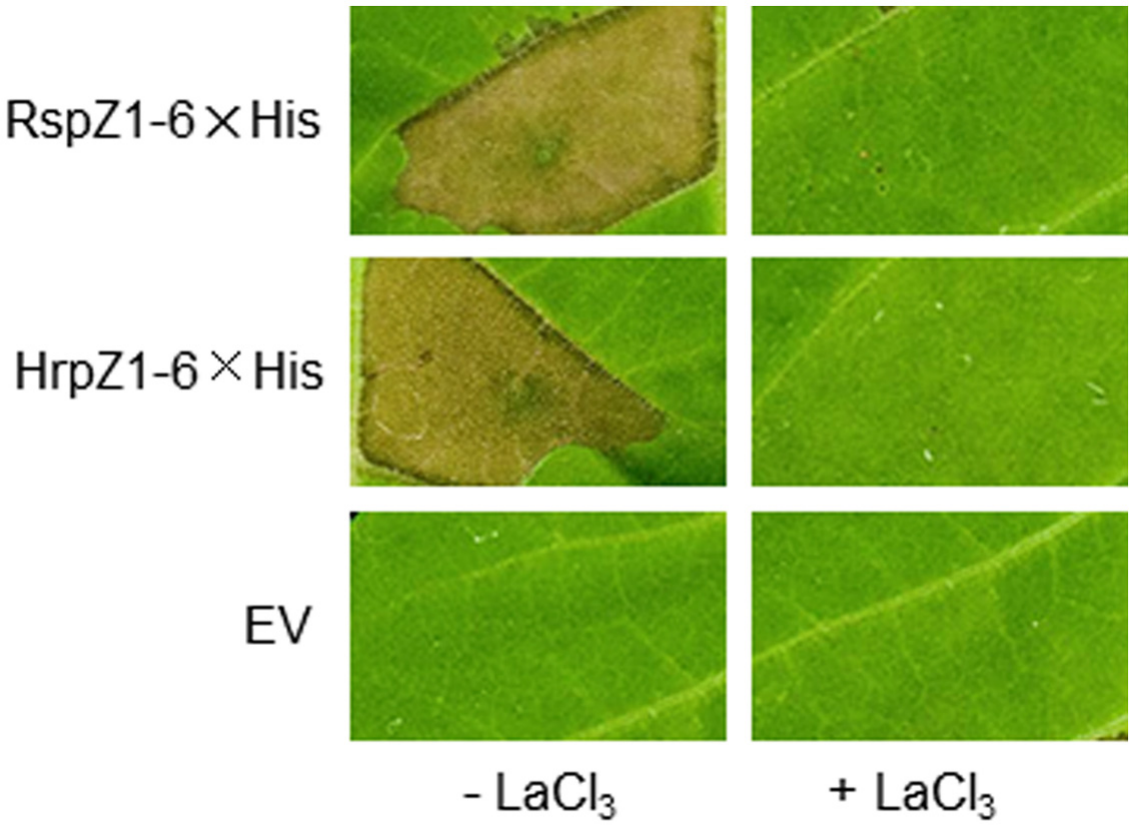
**Partially purified harpins RspZ1 (from *P. fluorescens* 2P24) and HrpZ1 (from *P. syringae* pv. *tomato* DC3000) elicit HR-like tissue collapse in tobacco leaves**. 6× His-tagged recombinant protein preparations were diluted in MES buffer and infiltrated into panels of tobacco leaves with or without 2 mM lanthanum chloride, an inhibitor of HR. The presence or absence of confluent cell death was evaluated 24 h post-infiltration. A mock, undiluted, empty-vector (EV) preparation was always tested in parallel. The experiment was repeated twice with similar results.

It is known that the primary property of harpins is their ability to elicit cell death when infiltrated into tobacco leaf intercellular spaces. To test for cell death elicitation, possibility by RspZ1, which is a new harpin candidate in this work, we produced RspZ1 of strain 2P24 from *E*. *coli* cells carrying an appropriate derivative of pET-22b (Supplementary Figure S2). Here, HrpZ1 from *P. syringae* DC3000 was employed as a positive control. Like HrpZ1, RspZ1 was soluble after overexpression in *E*. *coli*. Each protein at 100 μg/ml was infiltrated into leaves of tobacco with or without lanthanum chloride, an inhibitor of harpin-induced cell death (He et al., 1993; Kvitko et al., 2007). Surprisingly, RspZ1 elicited a lanthanum chloride-inhibitable cell death in tobacco, which was the same as HrpZ1 (**Figure 4**). An undiluted empty-vector preparation infiltrated into the same leaf as a control could not elicit any visible response. Elicitation of cell death by the application of an exogenous protein indicated that RspZ1from *P. fluorescens* 2P24 possesses a defining property of harpin proteins.

### The T3SS of *P. fluorescens* 2P24 Retains Ability to Secrete Effectors, but Loses the Effector Translocation Trait

**FIGURE 5:**
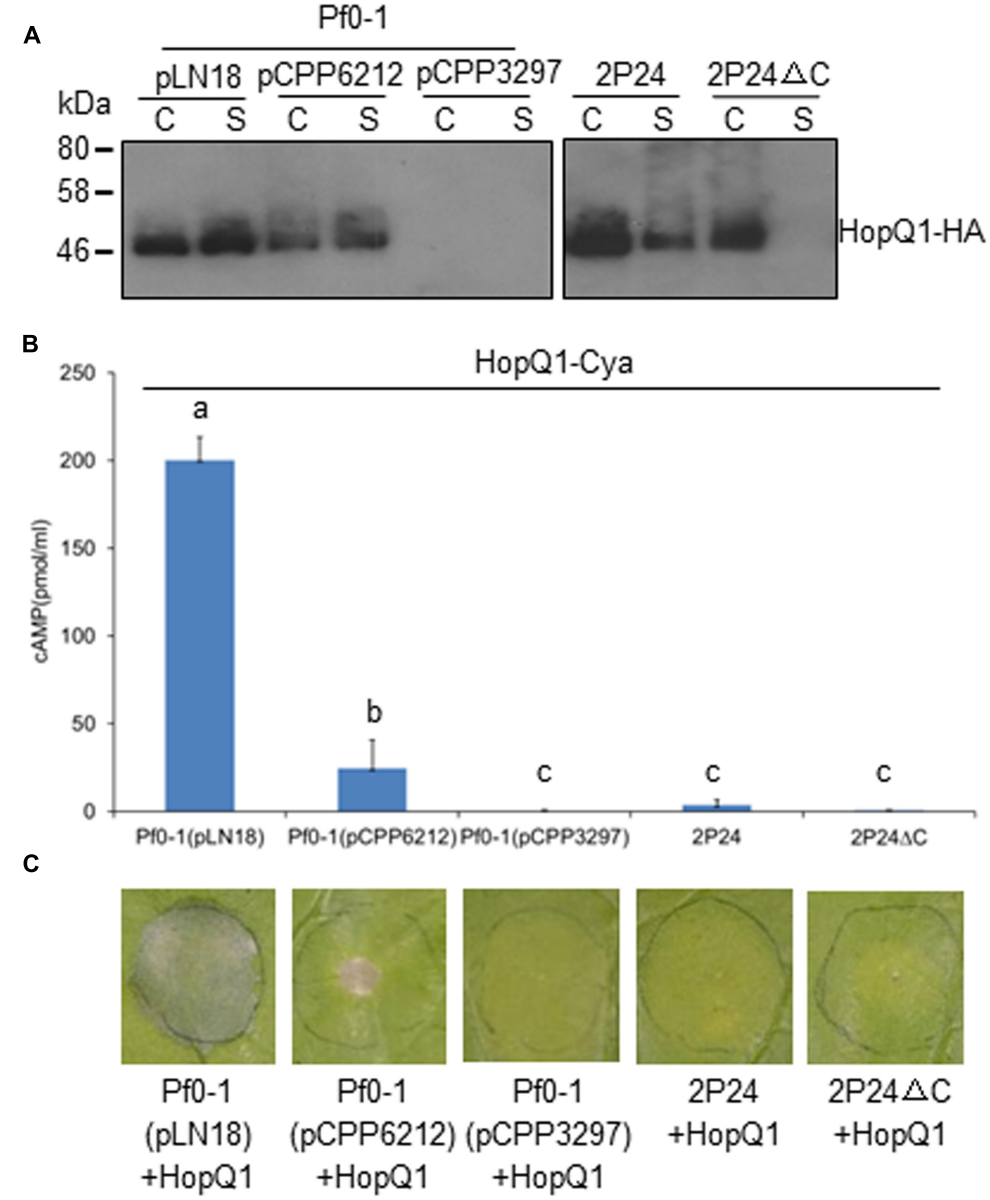
***P. fluorescens* 2P24 has a T3SS-dependent secretion^+^/translocation^-^ capacity. (A)** HopQ1-HA secretion assay. Strains transformed with pCPP5372-hopQ1 were grown from an OD_600_ of 0.3 to an OD_600_ of 0.5. Cell pellet (C) and supernatant (S) fractions were separated by centrifugation. Fractions were analyzed by SDS-PAGE, followed by immunoblotting with anti-HA antibodies. **(B)** HopQ1-CyaA translocation into *N. benthamiana*. Strains transformed with pCPP5371-hopQ1 were infiltrated into panels of *N. benthamiana* leaves at 10^8^ cfu/ml. 1.0 cm leaf disks were collected at 12 h post-inoculation. Effector translocation assays, based on cAMP production by the HopQ1-CyaA reporter, were performed in triplicate on disks from independent leaves. Means shown with the same letters are not different statistically at the 5% confidence level on the basis of Duncan’s multiple range test. **(C)** Strain 2P24 expressing HopQ1 in *N. benthamiana* does not elicit tissue collapse in accordance with the failure of translocation in 2P24. The bacteria carrying pCPP5372-hopQ1 were infiltrated as described above. Cell death was evaluated and representative leaves were photographed 48 h after inoculation. The experiment was repeated three times with similar results.

T3SSs are complex macromolecular machines that span both the bacterial cell envelope and host cell barriers. The basic property of T3SS is to secrete and deliver proteins, commonly termed effectors, from the bacterial cytoplasm into the host cytoplasm (Alfano and Collmer, 1997; Xin and He, 2013). To determine whether the T3SS of strain 2P24 has the natural traits of T3SS, we used hemagglutinin (HA) immunoblot analysis and an CyaA injection assay to test HopQ1 secretion and translocation in 2P24. We introduced constructs carrying HopQ1, an avirulence determinant of *P. syringae* DC3000 in *N. benthamiana* (Wei et al., 2007), into 2P24, 2P24ΔC, Pf0-1, and its derivatives. The strains were grown overnight in KB broth, and cell-bound and supernatant fractions were subjected to Western blot. HopQ1-HA was detected in supernatant fractions from wild type 2P24, but not from the 2P24 *rscC* mutant (**Figure 5A**). Meanwhile, HopQ1-HA was detected also in supernatant fractions from Pf0-1with pLN18 and pCPP6212 (Oh et al., 2010), which is a pLN18 derivative missing *hrpK1*and a key translocator encoding gene; HopQ1-HA was not detected from Pf0-1 with pCPP3297, a pLN18 *hrcC* mutant defective in type III secretion (**Figure 5A**). These results indicated that effector proteins are secreted in culture through *P. fluorescens* T3SS.

We then infiltrated the strains harboring HopQ1-CyaA into *N. benthamiana* leaves and determined cAMP levels, a product of the CyaA-catalyzed reaction, and cell deaths 12 and 48 h later, respectively. Plant tissue inoculated with *P. fluorescens* Pf0-1(pLN18)-expressing HopQ1-CyaA showed high levels of cAMP and strong HR, but we detected a sharply lower level of cAMP from the leaves with *P. fluorescens* Pf0-1(pCPP6212)-expressing HopQ1-CyaA, and no cAMP was detected from the leaves with *P. fluorescens* Pf0-1(pCPP3297)-expressing HopQ1-CyaA (**Figure 5B**). Accordingly, HopQ1-CyaA expressed in *P. fluorescens* Pf0-1(pLN18), but not in *P. fluorescens* Pf0-1(pCPP3297), triggered very strong cell death (**Figure 5C**). *P. fluorescens* Pf0-1(pCPP6212)-expressing HopQ1-CyaA elicited a weak cell death, because of the missing key translocator HopK1 (**Figure 5C**). However, we did not detect cAMP and cell death from plant tissue where HopQ1-CyaA was expressed in wild type 2P24 and 2P24ΔC (**Figures 5B,C**). Taken together, the results demonstrated that HopQ1 was injected into plant leaf cells by *P. syringae*, but not by *P. fluorescens* T3SS.

### The T3SS Mutation has no Changes on Biocontrol Capacity of Strain 2P24

The *rscC* mutant strain was tested *in vitro* for possible phenotypic changes in traits that contribute to biocontrol by *P. fluorescens* 2P24. The mutant was compared to the parental strain 2P24 for the ability to produce secondary metabolites, such as the antibiotic 2,4-DAPG, HCN, exoprotease, and siderophores. Unfortunately, no phenotypic changes were found from the *rscC* mutant in any of the aforementioned tests (data not shown). The mutant strain was tested also for the ability to antagonize the plant pathogens *Rhizoctonia solani* and *Ralstonia solanacearum*. The results indicated that inactivation of the T3SS did not reduce the antagonistic ability of strain 2P24 against plant pathogens (Supplementary Table S1). The further biocontrol assays on tomato bacterial wilt disease also showed that the T3SS mutation did not result in a reduction in the biocontrol activity of strain 2P24 (Supplementary Table S2).

### Strain 2P24 can Trigger Strong Functional MTI that is not Associated with T3SS

**FIGURE 6:**
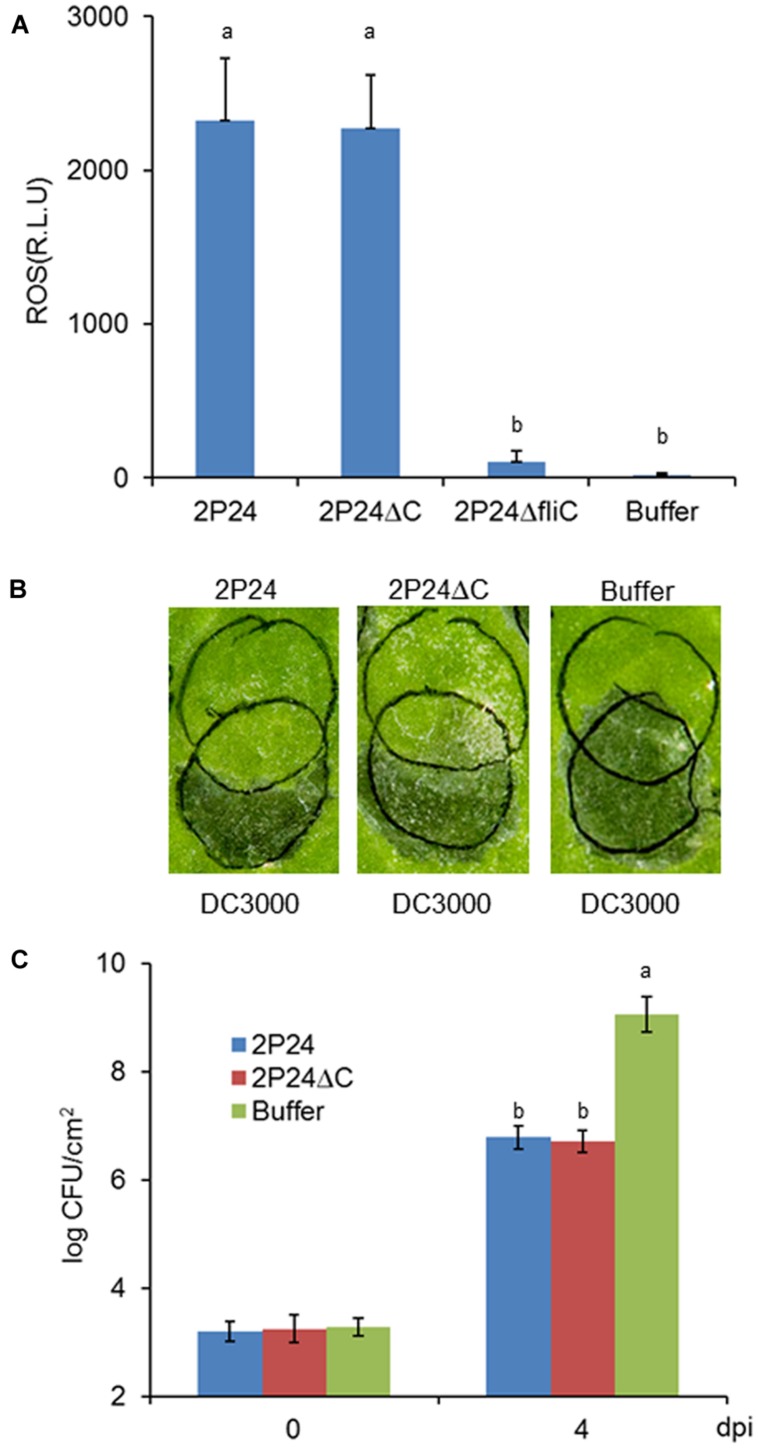
***P. fluorescens* 2P24 and the *rscC* mutant have same strong functional MTI in *N. benthamiana* as determined by multiple assays. (A)** ROS assay of strain 2P24 and the *rscC* mutant. The bacteria were infiltrated at 10^8^ cfu/ml into *N. benthamiana* leaves and 15 h later assayed for ROS production using L-102 chemiluminescence. ROS assay results are presented as the mean and SD based on three samples from three different plants. Means shown with the same letters are not different statistically at the 5% confidence level on the basis of Duncan’s multiple range test. **(B)** Challenge inoculum HR assays for functional MTI. Strain 2P24 and the *rscC* mutant were initiated by infiltrating *N. benthamiana* leaves with 10^8^ cfu/ml (upper circles). After 6 h, an overlapping inoculation of 10^7^ cfu/ml of the HR-inducing strain *P. syringae* pv. *tomato* DC3000 (lower circles) was made. The presence or absence of confluent cell death in the overlapping region was evaluated 48 h after strain DC3000 infiltration. **(C)** In the assay for functional MTI based on relative growth of virulent bacteria, strain 2P24 and the *rscC* mutant were infiltrated into *N. benthamiana* leaves at 10^8^ cfu/ml and challenged 6 h later with an overlapping inoculation of DC3000ΔhopQ1 at 10^5^ cfu/ml. At the indicated days after the challenge inoculation (dpi), 0.5 cm leaf disks were collected from areas of overlap between pre-treatment and challenge inoculations and bacterial populations were determined by dilution plating. Bacterial populations from three independent leaves were assayed in triplicate. Means shown with the same letters are not different statistically at the 5% confidence level on the basis of Duncan’s multiple range test.

*Pseudomonas fluorescens* 2P24 is a soil-born PGPR that is present normally in the rhizosphere. We did not know if it could trigger local MTI when inoculated in plant leaves or whether the T3SS had some effect on potential MTI. We then used established assays to determine the extent that 2P24 and 2P24ΔC trigger ROS production, suppress *P. syringae* DC3000 HR and DC3000Δ*hopQ1* disease as reported previously (Wei et al., 2013). Strain 2P24 triggered high levels of ROS production that might be induced by the flagellin because the *fliC* mutant of 2P24 failed to trigger ROS (**Figure 6A**), which is consistent with a previous report (Wei et al., 2013). Strain 2P24 also exhibited strong suppression of HR triggered by *P. syringae* DC3000 and pathogen growth of *P. syringae* DC3000Δ*hopQ1* in *N. benthamiana* (**Figures 6B,C**). However, the deficiency of the T3SS did not impact functional MTI elicited by 2P24 in any of these tests (**Figure 6**).

## Discussion

Most of the *P. fluorescens* bacteria are natural inhabitants of plant roots and soil. An increasing number of *P. fluorescens* strains are being studied due to their ability to produce secondary metabolites, such as antibiotics, volatile compounds, HCN, siderophores, cell wall degrading enzymes, and phytohormones (Majumder et al., 2014). As part of our research into the genetic basis for biocontrol capacity of *P. fluorescens* strain 2P24, we isolated and identified a gene cluster that encoded a typical T3SS from the genomic library. The T3SS gene cluster of *P. fluorescens* 2P24, *rsp/rsc*, showed greatest similarity to the *hrp/hrc* pathogenicity island of *P. syringae*, both at the level of genomic organization and amino acid sequence. The *rsp/rsc* cluster contained all of the nine conserved *hrc* genes and the *hrpA1* gene, which composes the basal body and pilus of the T3SS in plant pathogens (Tampakaki et al., 2004). The same composition and organization of T3SS genes were found also from *P. fluorescens* Q8r1-96 (Mavrodi et al., 2011). And yet, *P. fluorescens* Q8r1-96 and 2P24 have the most intact T3SS components compared to other *P. fluorescens* strains.

Although the T3SS in *P. fluorescens* has been studied for many years, its supramolecular structure is still obscure. The presence of *hrpA1* and all *hrc* homologs lends support to the proposition that T3SS machinery is produced in *P. fluorescens* 2P24. In this study, we modified the procedures and then isolated and visualized successfully the T3SS complexes from strain 2P24 for the first time after many attempts. Because *P. fluorescens* 2P24 also produces flagella, which is another kind of surface appendage that is very difficult to distinguish from the T3SS pilus when detached from bacteria, we created and used a *fliC* mutant that does not produce flagellin and forfeits swimming ability, under the guidance of similar reports on *P. syrinage* and *Salmonella typhimurium* (Kubori et al., 1998; Hu et al., 2001). The secretion machinery of *P. fluorescens* 2P24 exhibited a pilus-like structure similar to that of *P. syringae* that is composed of an elongated pilus and a cylindrical basal body (Hu et al., 2001). The successful isolation and identification of T3SS machinery from *P. fluorescens* 2P24 confirms the integrity of *rsp/rsc* genes in the genomic organization. Unlike the isolation condition for T3SS complex from plant pathogen *P. syringae* (Roine et al., 1997), we only were able to isolate the T3SS complex from KB rich medium, but not from the *hrp*-inducing medium. This might suggest that the T3SS in *P. fluorescens* 2P24 is not a strict pathogenic *hrp*-induced system, which is consistent with the presence of PGPR in the rhizosphere but not the leaf ecosystems.

Bacterial T3SS is of special interest because, by utilizing this system, bacteria are able to inject bacterial effector proteins directly into host cells. The typical process includes crossing the bacterial membranes termed secretion and the step of crossing the host membranes being called translocation (Alfano and Collmer, 2004). The cosmid pLN18 that contains a functional cluster of *hrp/hrc* T3SS genes from *P. syringae*, which is expressed in *P. fluorescens*, is sufficient to deliver effector proteins into plant cells (Huang et al., 1988). To test the functionality of *P. fluorescens* 2P24 T3SS, we introduced HopQ1 from *P. syringae* into *P. fluorescens* 2P24 and Pf0-1(pLN18) and tried to observe a HopQ1-dependent HR in leaves of *N. benthamiana*. However, we did not find expected HR from 2P24-expressing HopQ1, but we did find HR from *P. fluorescens* Pf0-1(pLN18)-expressing HopQ1. An immunoblot analysis showed that HopQ1-HA was secreted successfully through the T3SS by strain 2P24 that was dependent on the *rscC* gene present. But the HopQ1-CyaA translocation assay demonstrated that strain 2P24 failed to inject HopQ1-CyaA into plant cells, although a high level of HopQ1-CyaA proteins was detected from plant leaves expressed by *P. fluorescens* Pf0-1(pLN18). Further determination suggested the HopQ1-dependent HR expressed by *P. fluorescens* Pf0-1(pLN18) was patently dependent on HrpK1, which is a major translocator of bacterial T3SS, and which is absent from strain 2P24. Although the most similar T3SS in *P. fluorescens* Q8r1-96 was reported to translocate Rop effectors into tobacco leaves, the level of effector proteins was 100-fold lower than that expressed by *P. fluorescens* 55(pLN1965) (Mavrodi et al., 2011). It is very difficult to postulate that such translocation capacity by *P. fluorescens* Q8r1-96 is sufficient to trigger strong effector-dependent phenotypes like HR. Mavrodi et al. (2011) showed that the three Q8r1-96 Rop effectors expressed in *P. fluorescens* 55(pHIR11) and *P. fluorescens* 55(pLN1965) were capable of suppressing HopA1-dependent HR and flg22-dependent ROS production. It should be noted that these capabilities were based on high translocation levels of pHIR11and (pLN1965) in *P. fluorescens* 55, but not in the native T3SS of *P. fluorescens* Q8r1-96 (Mavrodi et al., 2011). Although the genome of strain 2P24 has not been sequenced and we do not know if 2P24 really has effector homologs, the fact that wild type 2P24 and the *rscC* mutant triggered the same high level of ROS production suggested that no significant T3SS-dependent MTI suppressor was present in strain 2P24. We also found that 2P24-expressing HopQ1 did not reduce the ROS level of wild type 2P24 (Supplementary Figure S3), which indicated also that HopQ1 was not translocated into plant cells through the T3SS of strain 2P24.

Besides effectors, other proteins like harpins secreted by T3SS play a critical role in microbe-host interactions. Harpins are universal components of plant pathogen T3SSs, which can elicit innate immunity when applied exogenously to plant cells (He et al., 1993; Kvitko et al., 2007). In *P. syringae* DC3000, four harpin proteins, HrpZ1, HrpW1, HopAK1, and HopP1, triggered cell death in tobacco with the purified proteins and they were involved also in effector translocation (Kvitko et al., 2007). The DNA sequence of the *P. fluorescens* 2P24 *rsp/rsc* cluster revealed the presence of a harpin-like encoding gene *rspZ1*. The purified RspZ1 protein elicited cell death in tobacco as do the usual harpin proteins. And *rspZ1* also complemented the mutation of *hrpZ1* from pHIR11 to translocate HopA1 and trigger cell death in tobacco. To date, in all of T3SS^+^ PGPR, only *P. fluorescens* Q8r1-96 was reported as having a RspZ1 homolog in the *rsp/rsc* cluster, whereas the functionality of it has not been determined (Mavrodi et al., 2011). Although our work could not define HrpZ1 as the sole harpin and translocator in strain 2P24, our results show for the first time that the cell death-like innate immunity triggered by RspZ1 aids our comprehensive understanding of the induced immunity and resistance by PGPR.

MAMPs are common elicitors in PGPR for basic resistance and induced systemic resistance (ISR) in plants (De Vleesschauwer and Hofte, 2009). Flagellin was investigated as the major MAMPs in pathogenic *P. syrinage* DC3000 or non-pathogenic *P. fluorescens* Pf0-1 (Wei et al., 2013). The flagellin-triggered MTI could be suppressed by injection of effectors (Wei et al., 2013). In *P. fluorescens* Q8r1-96, RopM, RopB, and RopAA suppressed both MTI and ETI immune responses, which was contrary to the capacity of Q8r1-96 to induce ISR in *Arabidopsis thaliana* (Mavrodi et al., 2011). However, strain 2P24 could elicit strong functional MTI and its endogenous TTSS could not deliver effectors to suppress MTI and ETI (**Figure 6** and Supplementary Figure S3), which is apparently consistent with the capacity of strain 2P24 to induce resistance in tomato (Wei et al., 2004a). Summarily, our data suggest that *P. fluorescens* 2P24 is a fantastic PGPR agent, which not only produces important secondary metabolites, but which also elicits strong functional MTI. Although we were unable to identify a role for 2P24 T3SS in biocontrol, it does not mean the T3SS in PGPR has no biological role. As a rhizosphere colonization bacterium, 2P24 is not like phyllosphere-derived pathogens in essence to inject proteins into mesophyll cells and trigger local innate immunity. A plausible explanation is that the T3SS acquirement in PGPR strains might be a consequence of cooption and could play a role in rhizosphere environment adaption and fitness (Jackson et al., 2005). Our ability to isolate the secretion^+^/translocation^-^ T3SS machinery clarifies the basic functionality of PGPR T3SS, which should focus our future attention on identifying potential T3SS-secreted proteins that occur during root colonization or cell–cell interactions.

## Author Contributions

H-LW and XZ designed research; PL, WZ, and H-LW performed research; PL, WZ, L-QZ, and HW analyzed data; H-LW wrote the paper.

## Conflict of Interest Statement

The authors declare that the research was conducted in the absence of any commercial or financial relationships that could be construed as a potential conflict of interest.
