# Supplementary material for: Supramolecular Structure and Functional Analysis of the Type III Secretion System in Pseudomonas fluorescens 2P24
Source: Front Plant Sci. 2016 Jan 5;6:1190. doi: 10.3389/fpls.2015.01190 (PMC4700148; doi:10.3389/fpls.2015.01190)
Supplement: Supplementary file 1 [file Data_Sheet_1.DOCX]

Supplementary Material

Supramolecular structure and functional analysis of the type III secretion system in *Pseudomonas fluorescens* 2P24

**Ping Liu1, Wei Zhang3, Li-Qun Zhang2, Xingzhong Liu1, Hai-Lei Wei1***

*** Correspondence:** Corresponding Author: weihl@im.ac.cn

# Supplementary Figures and Tables

##
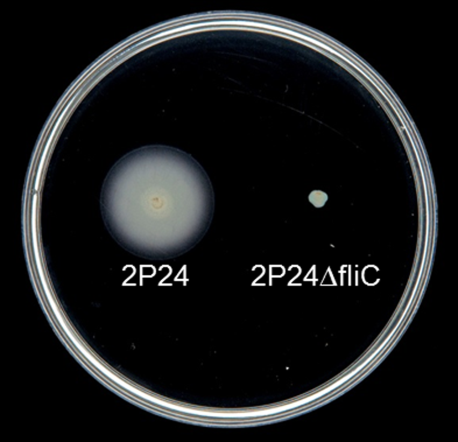
Supplementary Figures

##
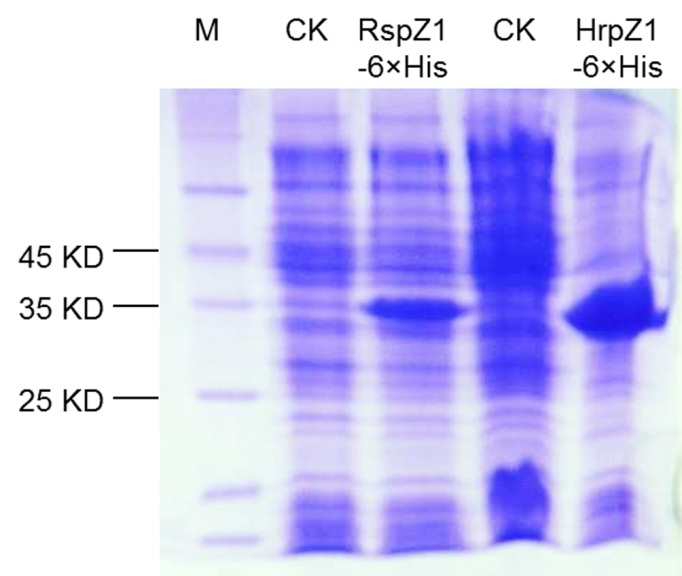
Supplementary Figure 1. The *fliC* mutant *P. fluorescens* 2P24 is deficient in motility on semisolid agar plates. Cells were inoculated with a toothpick from an overnight KB agar plate onto a swim plate (KB plus 0.3% agar) and photographed after 24 h incubation at 30ºC.

**Supplementary Figure 2.** Purification of the His-tag recombinant harpin proteins expressed in *E. coli*. SDS-PAGE analysis of the eluted fractions during the affinity purification procedure after coomasie staining. Molecular markers are on the left.

**
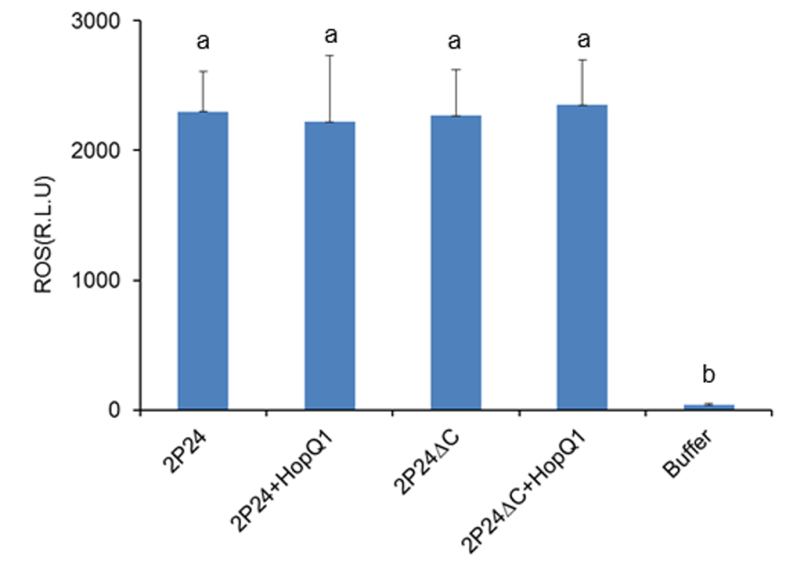
**

**Supplementary Figure 3.** ROS assay of HopQ1 expressed in strain 2P24 and the *rscC* mutant. The bacteria were infiltrated at 10^8^ cfu/ml into *N. benthamiana* leaves and 15 h later assayed for ROS production using L-102 chemiluminescence. ROS assay results are presented as the mean and SD based on three samples from three different plants. Means shown with the same letters are not statistically different.

## Supplementary Tables

**Supplementary Table 1.** **Antagonistic ability of *Pseudomonas fluorescens* 2P24 and its *rscC* mutant**

|  | 2P24 | 2P24∆C |
| --- | --- | --- |
| *Rhizoctonia solani* | 0.81±0.08 | 0.80±0.11 |
| *Ralstonia solanacearum* | 0.75±0.12 | 0.76±0.09 |

**Supplementary Table 2. Control efficiency of *P. fluorescens* 2P24 and its *rscC* mutant on tomato bacterial wilt**

|  | Disease index | Control efficiency (%) |
| --- | --- | --- |
| CK | 84.3 | ─ |
| 2P24 | 25.7 | 69.5 |
| 2P24∆C | 24.9 | 70.4 |
